# Oral Tori in Chronic Peritoneal Dialysis Patients

**DOI:** 10.1371/journal.pone.0156988

**Published:** 2016-06-08

**Authors:** Chia-Lin Hsu, Ching-Wei Hsu, Pei-Ching Chang, Wen-Hung Huang, Cheng-Hao Weng, Huang-Yu Yang, Shou-Hsuan Liu, Kuan-Hsing Chen, Shu-Man Weng, Chih-Chun Chang, I-Kuan Wang, Aileen I. Tsai, Tzung-Hai Yen

**Affiliations:** 1 Department of Pediatric Dentistry, Chang Gung Memorial Hospital, Linkou, Taiwan; 2 Department of Nephrology, Chang Gung Memorial Hospital and College of Medicine, Chang Gung University, Linkou, Taiwan; 3 Kidney Research Center, Chang Gung Memorial Hospital, Linkou, Taiwan; 4 Department of Clinical Pathology, Far Eastern Memorial Hospital, New Taipei, Taiwan; 5 Department of Nephrology, China Medical University Hospital and College of Medicine, China Medical University, Taichung, Taiwan; 6 Center for Tissue Engineering, Chang Gung Memorial Hospital, Linkou, Taiwan; Hospital Universitario de La Princesa, SPAIN

## Abstract

**Background:**

The pathogenesis of oral tori has long been debated and is thought to be the product of both genetic and environmental factors, including occlusal forces. Another proposed mechanism for oral tori is the combination of biomechanical forces, particularly in the oral cavity, combined with cortical bone loss and trabecular expansion, as one might see in the early stages of primary hyperparathyroidism. This study investigated the epidemiology of torus palatinus (TP) and torus mandibularis (TM) in peritoneal dialysis patients, and analyzed the influences of hyperparathyroidism on the formation of oral tori.

**Method:**

In total, 134 peritoneal dialysis patients were recruited between July 1 and December 31, 2015 for dental examinations for this study. Patients were categorized into two subgroups based on the presence or absence of oral tori. Demographic, hematological, biochemical, and dialysis-related data were obtained for analysis.

**Results:**

The prevalence of oral tori in our sample group was high at 42.5% (57 of 134), and most patients with oral tori were female (61.4%). The most common location of tori was TP (80.7%), followed by TP and TM (14.0%), then TM (5.3%). All 54 TP cases were at the midline, and most were <2 cm (59.3%), flat (53.7%), and located in the premolar region (40.7%). Of the 11 TM cases, all were bilateral and symmetric, mostly <2 cm (81.9%), lobular (45.4%), and located at premolar region (63.6%). Interestingly, patients with oral tori had slightly lower serum levels of intact parathyroid hormones than those without oral tori, but the difference was not statistically significant (317.3±292.0 versus 430.1±492.6 pg/mL, P = 0.126). In addition, patients with oral tori did not differ from patients without tori in inflammatory variables such as serum high sensitivity C-reactive protein levels (6.6±8.2 versus 10.3±20.2 mg/L, P = 0.147) or nutritional variables such as serum albumin levels (3.79±0.38 versus 3.77±0.45 g/dL, P = 0.790). Furthermore, there were no differences between patients with and without oral tori in dialysis adequacy (weekly Kt/V_urea_, 2.14±0.39 versus 2.11±0.33, P = 0.533; weekly creatinine clearance rate, 59.31±17.58 versus 58.57±13.20 L/1.73 m^2^, P = 0.781), or peritoneal membrane transporter characteristics (P = 0.098).

**Conclusion:**

Secondary hyperparathyroidism does not contribute to the formation of tori in peritoneal dialysis patients. Further studies are warranted.

## Introduction

A torus is a common exostosis found in the oral cavity. TP develops in the midline of the hard palate’s vault, while TM occurs along the lingual aspect of the mandible. [1] Tori are classified into four morphological types: flat, spindle, nodular, and lobular. [1] Because tori are non-symptomatic and painless, they are usually found by accident during oral examinations. The pathogenesis is multifactorial, including genetic factor (autosomal dominant trait) and environmental influence (masticatory stress), age, sex, regional factor, ethic factor, but no single cause is definitive. [2] Removal of the tori is not always necessary but is an alternative to oral function rehabilitation with removable prostheses or implants. Since tori are a non-pathological change, no surgical intervention was required in these patients. [3]

Taiwan has been recognized as an endemic area for kidney disease, with the highest incidence and prevalence rates of end-stage renal disease (ESRD) in the world. According to 2015 Annual Data Report of United States Renal Data System [4], Taiwan, the Jalisco region of Mexico, and the United States continue to report the highest incidence of treated ESRD (458, 421 and 363 per million population, respectively. Furthermore, the highest prevalence of treated ESRD was reported for Taiwan, Japan, and the United States (3138, 2411, and 2043 per million population, respectively). [4]

Renal osteodystrophy, which is most evident in dialysis patients, usually begins when kidney functions deteriorate. The spectrum of skeletal abnormalities seen in renal osteodystrophy is classified according to the state of bone turnover. Classic descriptions of the histologic abnormalities include high-turnover (osteitis fibrosa cystica), low-turnover (osteomalacia, adynamic bone disease), and low-to-high bone disease (mixed uremic osteodystrophy). [5] Osteitis fibrosa cystica is the result of the development of secondary hyperparathyroidism. [6] It is characterized by increased bone turnover, an increase in the number and activity of osteoblasts and osteoclasts, and variable amounts of peritrabecular fibrosis, which are increased osteoids with a woven pattern. Osteomalacia is characterized by increased osteoid seam width, increased in the trabecular surface area covered by osteoids, and decreased bone mineralization. Adynamic bone disease is characterized by normal or decreased osteoid volume and reduced bone formation rate, and reduced numbers of osteoblasts and osteoclasts. The gold standard for the diagnosis and specific classification of renal osteodystrophy is a bone biopsy with bone histomorphometry. [7] Nevertheless, most nephrologists do not perform bone biopsies on a routine basis.

In a study, Sisman et al [8] investigated the prevalence, size, location and shape of TP in 91 chronic peritoneal dialysis patients. The prevalence of TP was 41.7%. Most cases of TP were < 2 cm in size (81.6%) and spindle-shaped (78.9%). The duration of PD was statistically higher in patients with TP size > 2 cm (6.8±3.6 years) than patients with TP size of < 2 cm (3.5±2.6 years). Thus, the development of TP in peritoneal dialysis patients was attributed to an underlying disorder, such as secondary hyperparathyrodism [8]. On the other hand, Chao et al found that the prevalence of oral tori in 119 chronic hemodialysis patients was 33.6%. [9] The most common location of tori was TP (70.0%), followed by TM (20.0%), and then both TP and TM (10.0%). Of the 40 tori cases, most (67.5%) were <2 cm in size; moreover, the majority (52.5%) were flat in shape. Notably, the levels of intact parathyroid hormones in these hemodialysis patients did not differ in patients with or without tori (P = 0.611). Furthermore, patients with tori did not differ from patients without tori in inflammatory variables such as log high-sensitivity C-reactive protein (P = 1.000) or nutritional variables such as albumin (P = 0.247). Finally, there were no differences between patients with and without tori in adequacy of dialysis (P = 0.577). Therefore, neither hyperparathyroidism nor inflammation malnutrition syndrome was found to contribute to the formation of oral tori in chronic hemodialysis patients. [9]

The objective of this study was to undertake a broader assessment of potential environmental influences and, in doing so, address whether medical conditions, or chronic kidney disease—mineral and bone disease, or inflammation malnutrition syndrome were associated with chronic peritoneal dialysis patients with oral tori.

## Material and Methods

### Ethical statement

This clinical study was carried out in accordance with Declaration of Helsinki for Human Experimentation and was approved by the Medical Ethics Committee of Chang Gung Memorial Hospital. The Institutional Review Board numbers were 104-6913C and 102-2761B, and all patients provided written informed consent.

### Patients

All study patients were recruited between July 1 and December 31, 2015 from the Chang Gung Memorial Hospital, Linkou, Taiwan. Only patients undergoing chronic peritoneal dialysis for more than 6 months were enrolled, after excluding those with malignancies [10], active infectious diseases, hospitalizations, or surgery in the past 3 months, or lead [11] or cadmium [12] intoxication. The peritoneal dialysis prescription for each patient was based on the peritoneal membrane characteristics as determined by the peritoneal equilibration tests, with intermittent therapies used primarily for patients with high transport characteristics and continuous therapies for those with average or low transport characteristics. Low-calcium (1.5 or 1.25 mmol/L), icodextrin-based (7.5 g/dL) or standard dialysates containing glucose (sodium, 135 mmol/L; lactate, 35 mmol/L; calcium, 1.75 mmol/L) were used according to the patients' peritoneal transport characteristics and serum calcium levels to maintain adequate ultrafiltration and normal calcium levels. Dialysis prescription aimed at obtaining a total Kt/V of at least 1.8 per week.

### Groups

Patients who met the inclusion criteria were classified into 2 groups according to the presence or absence of oral tori.

### Laboratories

All laboratory values, including blood cell counts, biochemical data, dialysate/plasma creatinine ratio, peritoneal transport characteristics, weekly creatinine clearance, weekly Kt/V_urea_, were measured by automated and standardized methods. All blood samples were collected in the morning after at least 12 hours of fasting. Serum levels of albumin, blood urea nitrogen, creatinine and transferring saturation were measured and used as nutritional markers. Serum levels of calcium, phosphate, and intact parathyroid hormone were also measured and the corrected serum calcium level was calculated as: calcium (mg/dL) = [0.8 (4.0—albumin [g/dL])]. All other markers were measured via standard laboratory methods using an automatic analyzer.

### Diagnosis of oral tori

**Fig 1:**
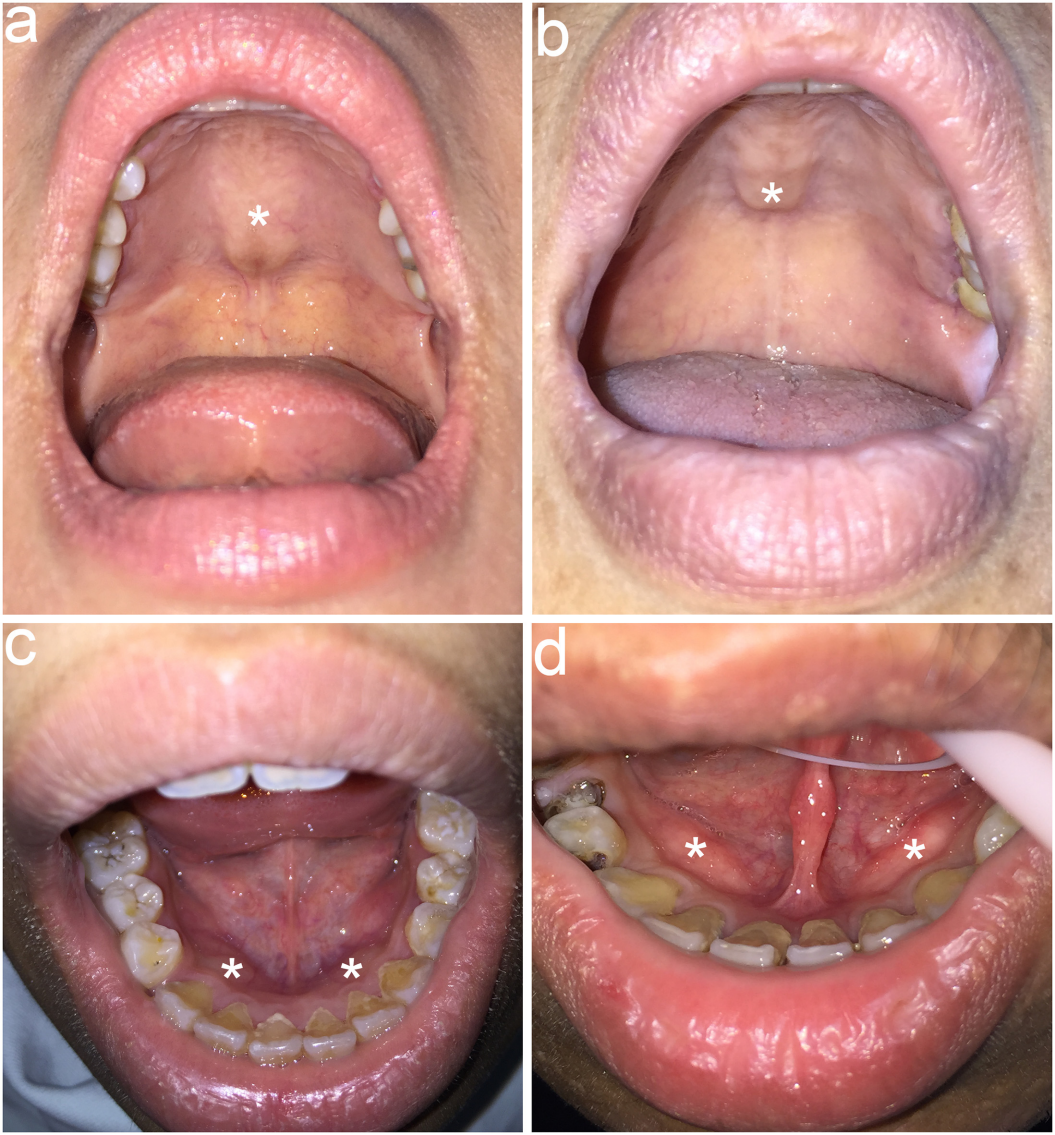
a, b. Torus palatinus. Intraoral view of two peritoneal dialysis patients with a flat torus palatinus (asterisk), which is an exophytic bony mass that arises along the midline of the hard palate. c, d. Torus mandibularis. Intraoral view of two peritoneal dialysis patients with a symmetrical lobular torus mandibularis (asterisk), which is an exophytic bony mass that arises along the lingual aspect of the mandible. Heavy dental calculus accumulating at cervical portion of teeth was also found in most patients.

The same dentist examined all patients and used mouth mirrors or tongue blades to check the oral condition of these patients. The examination for oral tori consisted of inspection and palpation. TP (Fig 1a and 1b) was defined as a raised bony exostosis along the midline of the hard palate whereas TM (Fig 1c and 1d) was defined as exostosis that develops along the lingual aspect of the mandible. The maximum elevation of the outgrowth of tori was used to measure the size of tori. Tori were graded as>2 cm or <2 cm using a periodontal probe, as described by Gorsky et al [13]. The shape of tori was classified as flat, spindle, nodular, or lobular according to the criteria described by Jainkittivong et al [14].

### Statistical analysis

Continuous variables were expressed as a mean with a standard deviation, while categorical variables were expressed as numbers and percentages in brackets. All data were tested for normality of distribution and equality of standard deviation before analysis. Comparisons between the 2 groups of patients were performed using Student’s t test for quantitative variables and Chi-square or Fisher’s exact tests for categorical variables. The criterion for significance was a 95% confidence interval to reject the null hypothesis. All analyses were performed using IBM SPSS Statistics Version 20.0.

## Results

A total of 134 ESRD patients were recruited into this study (Table 1). The prevalence of oral tori in our sample group was high at 42.5% (57 of 134). Most patients with oral tori were female (61.4%). Furthermore, there were no significant differences in baseline variables between patients with and without tori formations (P > 0.05).

**Table 1:** Baseline characteristics of peritoneal dialysis patients with or without oral tori (n = 134).

Variable	All patients (n = 134)	Patients with oral tori (n = 57)	Patients without oral tori (n = 77)	P value
Age, year	47.2±14.9	46.2±14.9	47.9±15.0	0.521
Female sex, n (%)	69 (51.5)	35 (61.4)	34 (44.2)	0.048*
Body mass index, kg/m2	23.0±4.4	23.5±5.2	22.6±3.7	0.264
Hypertension, n (%)	81 (60.4)	36 (63.2)	45 (58.4)	0.581
Diabetes mellitus, n (%)	24 (17.9)	8 (14.0)	16 (20.8)	0.314
Coronary artery disease, n (%)	11 (8.2)	6 (10.5)	5 (6.5)	0.400
Dialysis duration, months	60.5±43.3	62.9±43.1	57.3±43.7	0.455
Smoking habit, n (%)	14 (10.4)	4 (7.0)	10 (13.0)	0.264
Alcohol consumption, n (%)	1 (0.7)	0 (0)	1 (1.3)	0.388
Betel nut habit, n (%)	1 (0.7)	0 (0)	1 (1.3)	0.388

Note:

*P<0.05

Table 2 shows that patients with oral tori had slightly lower serum levels of intact parathyroid hormones than those without oral tori, but the difference did not reach statistical significance (317.3±292.0 versus 430.1±492.6 pg/mL, P = 0.126). Nevertheless, there were no significant differences in the serum levels of alkaline phosphatase (83.5±53.8 versus 91.3±61.8 U/L, P = 0.447), calcium (9.7±1.1 versus 9.6±1.0 mg/dL, P = 0.858), or phosphorus (5.7±1.4 versus 5.6±1.4, P = 0.463) between groups. In addition, patients with oral tori did not differ from patients without tori in inflammatory variables such as high sensitivity C-reactive protein (6.6±8.2 versus 10.3±20.2 mg/L, P = 0.147) or nutritional variables such as albumin (3.79±0.38 versus 3.77±0.45 g/dL, P = 0.790). Table 3 shows that there were no differences between patients with and without oral tori in term of dialysis adequacy (weekly Kt/V_urea_, 2.14±0.39 versus 2.11±0.33, P = 0.533; weekly creatinine clearance rate, 59.31±17.58 versus 58.57±13.20 L/1.73 m^2^, P = 0.781). Finally, there was no difference in the peritoneal transporter characteristics between patients with and without tori (P = 0.098).

**Table 2:** Laboratory findings of peritoneal dialysis patients with or without oral tori (n = 134).

Variable	All patients (n = 134)	Patients with oral tori (n = 57)	Patients without oral tori (n = 77)	P value
Blood urea nitrogen, mg/dL	64.8±18.8	65.1±21.9	64.6±16.3	0.883
Creatinine, mg/dL	13.1±3.5	13.4±3.6	12.8±3.3	0.398
Uric acid, mg/dL	7.0±1.4	7.2±1.5	6.8±1.3	0.112
Estimated glomerular filtration rate, mL/min/1.73 m^2^	3.9±1.5	3.7±1.1	4.1±1.7	0.112
Sodium, mEq/L	136.4±4.0	136.7±4.1	136.2±4.0	0.513
Potassium, mEq/L	4.0±0.7	4.0±0.6	4.0±0.8	0.612
Calcium, mg/dL	9.6±1.0	9.7±1.1	9.6±1.0	0.858
Inorganic phosphorus, mg/dL	5.6±1.4	5.7±1.4	5.6±1.4	0.463
Fasting glucose, mg/dL	107.0±41.7	109.8±47.1	104.9±37.3	0.504
Glycated hemoglobin, %	5.63±0.87	5.61±0.99	5.64±0.77	0.815
Albumin, g/dL	3.78±0.42	3.79±0.38	3.77±0.45	0.790
Alkaline phosphatase, U/L	88.0±58.5	83.5±53.8	91.3±61.8	0.447
Total cholesterol, mg/dL	187.4±45.2	188.8±48.3	186.3±43.1	0.744
High-density lipoprotein, mg/dL	42.9±13.6	43.9±12.1	42.1±14.6	0.454
Low-density lipoprotein, mg/dL	139.0±84.4	136.9±75.8	140.5±90.8	0.808
Triglyceride, mg/dL	184.9±180.6	200.7±196.1	173.2±168.5	0.385
Aspartate aminotransferase, U/L	21.8±9.4	20.6±7.5	22.7±10.6	0.180
Alanine aminotransferase, U/L	17.3±7.8	16.1±5.8	18.1±8.9	0.115
Intact parathyroid hormone, pg/mL	382.1±421.5	317.3±292.0	430.1±492.6	0.126
Iron, ug/dL	81.9±36.8	82.3±32.6	81.7±39.9	0.923
Total iron binding capacity, ug/dL	280.5±61.3	270.3±62.2	288.0±59.9	0.098
Ferritin, ng/mL	391.6±595.6	369.6±289.7	407.9±747.2	0.714
White blood cell count, 103/uL	7.7±2.6	7.7±2.5	7.8±2.8	0.792
Red blood cell count, 106/uL	3.5±0.6	3.4±0.5	3.6±0.6	0.118
Hemoglobin, g/dL	10.2±1.4	10.1±1.3	10.3±1.5	0.391
Hematocrit, %	30.4±4.3	29.9±4.2	30.8±4.3	0.229
Mean corpuscular volume, fL	86.6±7.1	87.1±5.8	86.3±7.9	0.462
Mean corpuscular hemoglobin, pg/Cell	29.1±2.7	29.4±2.2	28.9±3.0	0.213
Mean corpuscular hemoglobin concentration, gHb/dL	33.6±1.1	33.8±1.0	33.4±1.1	0.082
Red blood cell distribution width, %	14.5±1.5	14.3±1.1	14.7±1.7	0.147
Platelet count, 10^3^/uL	228.0±81.2	231.8±84.3	225.2±79.4	0.645
High sensitivity C-reactive protein, mg/L	8.7±16.3	6.6±8.2	10.3±20.2	0.147

Note: P<0.05

**Table 3:** Dialysis-related data of peritoneal dialysis patients with or without oral tori (n = 134).

Variable	All patients (n = 134)	Patients with oral tori (n = 57)	Patients without oral tori (n = 77)	P value
Dialysate/plasma creatinine	0.66 ± 0.11	0.65± 0.11	0.67± 0.11	0.224
Peritoneal equilibration test				0.098
High, n (%)	13 (9.7%)	3 (5.3%)	10 (13.0%)	
High average, n (%)	58 (43.3%)	21 (36.8%)	37 (48.0%)	
Low average, n (%)	55 (41.0%)	30 (52.6%)	25 (32.5%)	
Low, n (%)	8 (6.0%)	3 (5.3%)	5 (6.5%)	
Weekly Kt/V_urea_	2.12± 0.35	2.14 ± 0.39	2.11 ± 0.33	0.533
Weekly creatinine clearance rate, L/1.73 m^2^	58.88± 15.16	59.31 ± 17.58	58.57 ± 13.20	0.781

Note: Kt/V_urea_ is a number used to quantify peritoneal dialysis treatment adequacy. K dialyzer clearance of urea. t dialysis time. V volume of distribution of urea.

Table 4 shows that 57 patients were found to have oral tori; 46 patients had TP (80.7%); 3 patients had TM (5.3%), and 8 patients had both TP and TM (14%). Of the 54 TP cases, all were midline and most were <2 cm in size (59.3%), flat in shape (53.7%), and located at premolar region (40.7%). Of the 11 TM cases, all were bilateral and symmetric, and most were <2 cm in size (81.9%), lobular in shape (45.4%), and located at premolar region (63.6%).

**Table 4:** Clinical findings of oral tori (n = 57).

Variable		
Type		
TP, n (%)	46 (80.7)	
TM, n (%)	3 (5.3)	
TP+TM, n (%)	8 (14.0)	
Symmetric occurrence	TP (n = 54)	TM (n = 11)
Symmetric, n (%)	54 (100.0)	11 (100.0)
Size	TP (n = 54)	TM (n = 11)
<2cm, n (%)	32 (59.3)	9 (81.9)
>2cm, n (%)	22 (40.7)	2 (18.1)
Shape	TP (n = 54)	TM (n = 11)
Flat, n (%)	29 (53.7)	0 (0)
Spindle, n (%)	9 (16.7)	2 (18.2)
Nodular, n (%)	11 (20.3)	4 (36.4)
Lobular, n (%)	5 (9.3)	5 (45.4)
Location	TP (n = 54)	TM (n = 11)
Incisor, n (%)	1 (1.9)	0 (0)
Premolar, n (%)	22 (40.7)	7 (63.6)
Molar, n (%)	5 (9.3)	0 (0)
Incisor + premolar, n (%)	3 (5.5)	4 (36.4)
Premolar + molar, n (%)	18 (33.3)	0 (0)
Incisor + premolar +molar, n (%)	5 (9.3)	0 (0)

Note: TP torus palatinus, TM torus mandibularis

## Discussion

Few data are available regarding the prevalence rate of oral tori in dialysis patients; this is the first study examining the prevalence of oral tori in ESRD patients treated with peritoneal dialysis in Taiwan. Our data revealed that 57 out of 134 (42.5%) peritoneal dialysis patients had oral tori. TP generally occurs in 4.1–60.5% of the population, and TM from 1.4–38.2%; different studies have reported marked differences between various ethnic groups (Table 5). In chronic hemodialysis patients, our previous data [9] indicated that the prevalence rates of TP and TM were 23.5% and 6.7%, respectively. In chronic peritoneal dialysis patients, Sisman et al [8] reported that the prevalence rate of TP was 41.6%, but the rate of TM was not assessed.

**Table 5:** Comparison of prevalence rate of oral tori from different studies.

Study	Year	Geographic area	Sample size,	Population	TP, % (female)	TM, % (female)	Age, year
Eggen et al. [36]	1986	Norwegian	829	Nonuremic	32.8 (68.3)	27.5 (54.5)	
Reichart et al. [24]	1988	German	1317	Nonuremic	13.5 (60.1)	5.2 (24.6)	1->80
Reichart et al. [24]	1988	Thailand	947	Nonuremic	23.1 (70.8)	9.2 (56.3)	1->80
Haugen et al. [26]	1992	Norway	5000	Nonuremic	9.2 (67.6)	7.2 (49.0)	16–89
Eggen et al [30]	1994	Norwegian	1181	Nonuremic	38.4 (60.1)	12.7 (43.1)	
Gorsky et al. [13]	1996	Israel	1002	Nonuremic	21.0 (63.9)		4–40*
Al-Bayaty [3]	2001	India	667	Nonuremic	9.4 (73.0)	5.7 (55.2)	11–50
Bruce et al. [28]	2004	Ghana	926	Nonuremic	3.9 (74.2)	12.1 (55.2)	
Yildiz et al. [20]	2005	Turkey	1943	Nonuremic	30.9 (50.7)		5–15
Jainkittivong et al. [14]	2007	Thailand	1520	Nonuremic	60.5 (62.8)	32.2 (48.1)	10–60
Sisman et al. [21]	2008	Turkey	2660	Nonuremic	4.1 (81.8)		13–85
Yoshinaka et al. [19]	2010	Japan	664	Nonuremic	17.0 (80.5)	29.7 (46.7)	60–82
Simunkovic et al [31]	2011	Croatia	1679	Nonuremic	42.9 (54.8)	12.6 (52.6)	9–99
Hiremath et al. [25]	2011	Malaysia	65	Nonuremic	50.8 (90.9)	4.6 (66.7)	13–59
Sisman et al. [8]	2012	Turkey	91	Uremic	41.7 (55.3)		19–81
Sathya et al. [23]	2012	Malaysia	1532	Nonuremic	12.0 (64.7)	2.8 (65.1)	10->40
Chiang et al. [29]	2014	Taiwan	2050	Nonuremic	21.1 (76.2)	24.2 (52.1)	<18->65
Chao et al. [9]	2014	Taiwan	119	Uremic	23.5(56.2)	6.7 (41.7)	9–90
Present study	2015	Taiwan	135	Uremic	40.3(63.0)	8.2 (54.5)	9–81.7.9

Note: Note: TP torus palatinus, TM torus mandibularis

* radiographic study

Our finding, which shows that patients with oral tori had slightly (but statistically insignificant) lower serum levels of intact parathyroid hormones than those without oral tori (P = 0.126), opposes Sisman’s hypothesis [8], which attributes the high prevalence of TP in peritoneal dialysis to secondary hyperparathyroidism. However, blood levels of intact parathyroid hormone were not measured during the study. In our previous study [9], the blood levels of intact parathyroid hormone also did not differ between hemodialysis patients with or without oral tori (P = 0.611). In addition, since renal osteodystrophy presents a broad spectrum of histologic abnormalities (osteitis fibrosa cystica, osteomalacia, adynamic bone disease, or mixed uremic osteodystrophy, etc), it is difficult to determine whether uremic milieu can predict oral tori risk. Furthermore, without bone biopsy, the gold standard of diagnosis, the utility of blood intact parathyroid hormone concentration in the assessment of renal osteodystrophy remains controversial.

The pathogenesis of oral tori has long been debated and is generally thought to be multifactorial with genetic and environmental factors, including occlusal (biting) forces, contributing to their formation. Another proposed mechanism [15] for oral tori is the combination of biomechanical forces, particularly in the oral cavity, combined with cortical bone loss and trabecular expansion that result in an increased incidence of TM. This preferential loss of cortical bone and increased formation of trabecular bone usually occurs in the early stages of primary hyperparathyroidism. In a study, Padbury et al [15] demonstrated that patients with primary hyperparathyroidism were more likely to have oral tori and reductions in radicular lamina dura on dental radiographs. Subsequently, Rai et al [16] also reported that loss of lamina dura, a ground-glass appearance, and mandibular cortical width reduction were common in patients with primary hyperparathyroidism, and these findings were significantly correlated with elevated parathyroid hormone and alkaline phosphatase. Notably, none of the patients had TP [16]. Since renal osteodystrophy presents a broad spectrum of histologic abnormalities (osteitis fibrosa cystica, osteomalacia, adynamic bone disease, or mixed uremic osteodystrophy), it is difficult to determine if uremic milieu could predict oral tori risk. Furthermore, the utility of blood intact parathyroid hormone concentration in the assessment of renal osteodystrophy remains controversial without bone biopsy, the gold standard of diagnosis. [7]

Most of our peritoneal dialysis patients with oral tori were females (61.4%). The idiopathic TP is transmitted as an autosomal dominant trait, and it is believed that there may be a dominant type linked to the X chromosome. [17] In a study of 162 Lithuanian twins [18], TM were found in 56.8% and TP in 1.8% of the subjects and a calculation of heritability estimate also verifies dominant influence of genetic factor on the etiology of oral bony outgrowths. No significant difference was found between men and women in the prevalence of oral tori [18]. Females were more likely to have TP, from 5.7% to 70.5% [8, 9, 13, 14, 19–30]. Only one study showed a higher TP prevalence in males than in females. [31] In our study, a higher TP prevalence rate was found in females. The prevalence of TM is higher in males [9, 13, 14, 23–26, 28, 29, 31–33]; however, in Sathya [23] and our studies, higher TM prevalence rates were found in females.

Most studies found that the most common onset-age ranged from the third to the fourth decades of life. [24, 26, 28, 33] The increased production of tori was associated with age [29]. The present study investigated ESRD patients with an average age of 47.2 years old, older than subjects in previous research which usually recruited patients from school examinations [14, 20, 25], dental outpatient care [3, 13, 22–24, 29, 33], or residents in certain areas [21, 31, 34] with a more average distribution of different ages. However, the prevalence rates of TP and TM in this study did not differ from other studies. [19, 32, 35]

TP can be classified as flat, nodular, lobular or spindle-shaped [3, 14, 24, 26], and TM is usually nodular, unilateral or bilateral, and single or multiple. [3, 24, 26]. In previous studies, the most common shape of TP and TM was the flat and nodular type. [13, 35] In our study, the most common TP shape was flat (53.7%), while the most common TM shape was lobular type (45.4%). Other studies show that the most common shape is spindle. [8, 14, 24, 31]. The current data were similar to our previous findings in hemodialysis population [9], which may be due to the similar ethnic background and environment.

## Conclusion and Limitations

The prevalence of oral tori in our sample group was high at 42.5% (57 of 134), and most patients with oral tori were female (61.4%). Analytical results revealed that secondary hyperparathyroidism did not contribute to the formation of tori in peritoneal dialysis patients. Nevertheless, the current study is limited by a small sample size, short follow-up duration, lack of healthy controls, lack of protocol dental radiograph analysis, lack of histopathology analysis between spontaneous and peritoneal dialysis induced tori, and lack of histochemical or genetic analysis of TP samples for marker of renal osteodistrophy. Further studies are warranted.

## References

[1] Antoniades DZ, Belazi M, Papanayiotou P. Concurrence of torus palatinus with palatal and buccal exostoses: case report and review of the literature. Oral surgery, oral medicine, oral pathology, oral radiology, and endodontics. 1998;85(5):552–7. . 9619673 10.1016/s1079-2104(98)90290-6

[2] Garcia-Garcia AS, Martinez-Gonzalez JM, Gomez-Font R, Soto-Rivadeneira A, Oviedo-Roldan L. Current status of the torus palatinus and torus mandibularis. Med Oral Patol Oral Cir Bucal. 2010;15(2):e353–60. . 19767716

[3] Al-Bayaty HF, Murti PR, Matthews R, Gupta PC. An epidemiological study of tori among 667 dental outpatients in Trinidad & Tobago, West Indies. Int Dent J. 2001;51(4):300–4. . 11570546 10.1002/j.1875-595x.2001.tb00842.x

[4] United States Renal Data System. 2015 USRDS annual data report: Epidemiology of kidney disease in the United States. National Institutes of Health, National Institute of Diabetes and Digestive and Kidney Diseases, Bethesda, MD, 2015.

[5] Jorgetti V. Review article: Bone biopsy in chronic kidney disease: patient level end-point or just another test? Nephrology (Carlton). 2009;14(4):404–7. 10.1111/j.1440-1797.2009.01148.x . 19563382

[6] Miller PD. Chronic kidney disease and the skeleton. Bone Res. 2014;2:14044 10.1038/boneres.2014.44 26273531 PMC4472138

[7] Sprague SM, Bellorin-Font E, Jorgetti V, Carvalho AB, Malluche HH, Ferreira A, et al Diagnostic Accuracy of Bone Turnover Markers and Bone Histology in Patients With CKD Treated by Dialysis. Am J Kidney Dis. 2015 10.1053/j.ajkd.2015.06.023 . 26321176

[8] Sisman Y, Gokce C, Sipahioglu M, Tarim Ertas E, Unal A, Oymak O, et al Torus palatinus in end-stage renal disease patients receiving peritoneal dialysis: Does renal osteodystrophy play a role? J Dent Sci 2012, 7(2):154–158.

[9] Chao PJ, Yang HY, Huang WH, Weng CH, Wang IK, Tsai AI, et al Oral tori in chronic hemodialysis patients. Biomed Res Int. 2015;2015:897674 10.1155/2015/897674 25918724 PMC4396140

[10] Wang TY, Hu CJ, Kuo CW, Chen Y, Lin JL, Yang CW, et al High incidence and recurrence of transitional cell carcinoma in Taiwanese patients with end-stage renal disease. Nephrology (Carlton). 2011;16(2):225–31. Epub 2011/01/29. 10.1111/j.1440-1797.2010.01366.x . 21272136

[11] Lin JL, Lin-Tan DT, Chen KH, Hsu CW, Yen TH, Huang WH, et al Blood lead levels association with 18-month all-cause mortality in patients with chronic peritoneal dialysis. Nephrol Dial Transplant. 2010;25(5):1627–33. Epub 2009/12/25. gfp663 [pii] 10.1093/ndt/gfp663 . 20031932

[12] Hsu CW, Lin JL, Lin-Tan DT, Huang WH, Chen KH, Yen TH. Association between blood cadmium levels and malnutrition in peritoneal dialysis. BMC Nephrol. 2014;15:17 10.1186/1471-2369-15-17 24428882 PMC3898399

[13] Gorsky M, Raviv M, Kfir E, Moskona D. Prevalence of torus palatinus in a population of young and adult Israelis. Arch Oral Biol. 1996;41(6):623–5. . 8937655 10.1016/0003-9969(96)00149-5

[14] Jainkittivong A, Apinhasmit W, Swasdison S. Prevalence and clinical characteristics of oral tori in 1,520 Chulalongkorn University Dental School patients. Surg Radiol Anat. 2007;29(2):125–31. 10.1007/s00276-007-0184-6 . 17340055

[15] Padbury AD Jr, Tozum TF, Taba M Jr, Ealba EL, West BT, Burney RE, et al The impact of primary hyperparathyroidism on the oral cavity. J Clin Endocrinol Metab. 2006;91(9):3439–45. 10.1210/jc.2005-2282 . 16822829

[16] Rai S, Bhadada SK, Rattan V, Bhansali A, Rao DS, Shah V. Oro-mandibular manifestations of primary hyperparathyroidism. Indian J Dent Res. 2012;23(3):384–7. 10.4103/0970-9290.102236 . 23059578

[17] Gorsky M, Bukai A, Shohat M. Genetic influence on the prevalence of torus palatinus. Am J Med Genet. 1998;75(2):138–40. . 9450873 10.1002/(sici)1096-8628(19980113)75:2<138::aid-ajmg3>3.0.co;2-p

[18] Auskalnis A, Bernhardt O, Putniene E, Sidlauskas A, Andriuskeviciute I, Baseviciene N. Oral bony outgrowths: prevalence and genetic factor influence. Study of twins. Medicina (Kaunas). 2015;51(4):228–32. 10.1016/j.medici.2015.07.001 . 26424187

[19] Yoshinaka M, Ikebe K, Furuya-Yoshinaka M, Hazeyama T, Maeda Y. Prevalence of torus palatinus among a group of Japanese elderly. J Oral Rehabil. 2010;37(11):848–53. 10.1111/j.1365-2842.2010.02100.x . 20609055

[20] Yildiz E, Deniz M, Ceyhan O. Prevalence of torus palatinus in Turkish schoolchildren. Surg Radiol Anat. 2005;27(5):368–71. 10.1007/s00276-005-0003-x . 16075159

[21] Sisman Y, Ertas ET, Gokce C, Akgunlu F. Prevalence of torus palatinus in cappadocia region population of Turkey. Eur J Dent. 2008;2(4):269–75. 19212533 PMC2634781

[22] Sawair FA, Shayyab MH, Al-Rababah MA, Saku T. Prevalence and clinical characteristics of tori and jaw exostoses in a teaching hospital in Jordan. Saudi Med J. 2009;30(12):1557–62. . 19936420

[23] Sathya K, Kanneppady SK, Arishiya T. Prevalence and clinical characteristics of oral tori among outpatients in Northern Malaysia. J Oral Biol Craniofac Res. 2012;2(1):15–9. 10.1016/S2212-4268(12)60005-0 25756026 PMC3941621

[24] Reichart PA, Neuhaus F, Sookasem M. Prevalence of torus palatinus and torus mandibularis in Germans and Thai. Community Dent Oral Epidemiol. 1988;16(1):61–4. . 3422622 10.1111/j.1600-0528.1988.tb00557.x

[25] Hiremath VK, Husein A, Mishra N. Prevalence of torus palatinus and torus mandibularis among Malay population. J Int Soc Prev Community Dent. 2011;1(2):60–4. 10.4103/2231-0762.97704 24478956 PMC3894068

[26] Haugen LK. Palatine and mandibular tori. A morphologic study in the current Norwegian population. Acta Odontol Scand. 1992;50(2):65–77. . 1604967 10.3109/00016359209012748

[27] Eggen S, Natvig B. Relationship between torus mandibularis and number of present teeth. Scand J Dent Res. 1986;94(3):233–40. . 3461543 10.1111/j.1600-0722.1986.tb01758.x

[28] Bruce I, Ndanu TA, Addo ME. Epidemiological aspects of oral tori in a Ghanaian community. Int Dent J. 2004;54(2):78–82. . 15119797 10.1111/j.1875-595x.2004.tb00259.x

[29] Chiang ML, Hsieh YJ, Tseng YL, Lin JR, Chiang CP. Oral mucosal lesions and developmental anomalies in dental patients of a teaching hospital in Northern Taiwan. J Dent Sci 2014; 9: 69–77.

[30] Eggen S, Natvig B, Gasemyr J. Variation in torus palatinus prevalence in Norway. Scand J Dent Res. 1994;102(1):54–9. . 8153581 10.1111/j.1600-0722.1994.tb01153.x

[31] Simunkovic SK, Bozic M, Alajbeg IZ, Dulcic N, Boras VV. Prevalence of torus palatinus and torus mandibularis in the Split-Dalmatian County, Croatia. Coll Antropol. 2011;35(3):637–41. . 22053535

[32] Yoshinaka M, Ikebe K, Furuya-Yoshinaka M, Maeda Y. Prevalence of torus mandibularis among a group of elderly Japanese and its relationship with occlusal force. Gerodontology. 2014;31(2):117–22. 10.1111/ger.12017 . 23167776

[33] Eggen S, Natvig B. Concurrence of torus mandibularis and torus palatinus. Scand J Dent Res. 1994;102(1):60–3. . 8153582 10.1111/j.1600-0722.1994.tb01154.x

[34] Romanos GE, Sarmiento HL, Yunker M, Malmstrom H. Prevalence of torus mandibularis in Rochester, New York, region. N Y State Dent J. 2013;79(1):25–7. . 23513545

[35] Al Quran FA, Al-Dwairi ZN. Torus palatinus and torus mandibularis in edentulous patients. J Contemp Dent Pract. 2006;7(2):112–9. . 16685302

[36] Eggen S, Natvig B. Relationship between torus mandibularis and number of present teeth. Scand J Dent Res 1986;94(3):233–40. 3461543 10.1111/j.1600-0722.1986.tb01758.x

